# Highly Flexible and Broad-Range Mechanically Tunable All-Wood Hydrogels with Nanoscale Channels via the Hofmeister Effect for Human Motion Monitoring

**DOI:** 10.1007/s40820-022-00827-3

**Published:** 2022-03-29

**Authors:** Guihua Yan, Shuaiming He, Gaofeng Chen, Sen Ma, Anqi Zeng, Binglin Chen, Shuliang Yang, Xing Tang, Yong Sun, Feng Xu, Lu Lin, Xianhai Zeng

**Affiliations:** 1 College of Energy, Xiamen University, Xiamen, 361102 People’s Republic of China; 2 State Key Laboratory of Pulp and Paper-Making Engineering, South China University of Technology, Guangzhou, 510640 People’s Republic of China; 3 Beijing Key Laboratory of Lignocellulosic Chemistry, Beijing Forestry University, No. 35, Tsinghua East Road, Haidian, Beijing, 100083 People’s Republic of China

**Keywords:** Wood hydrogel, Hofmeister effect, Tunable mechanical strength, Flexible, Biodegradable

## Abstract

Wood-based hydrogel with a unique anisotropic structure is an attractive soft material, but the presence of rigid crystalline cellulose in natural wood makes the hydrogel less flexible. In this study, an all-wood hydrogel was constructed by cross-linking cellulose fibers, polyvinyl alcohol (PVA) chains, and lignin molecules through the Hofmeister effect. The all-wood hydrogel shows a high tensile strength of 36.5 MPa and a strain up to ~ 438% in the longitudinal direction, which is much higher than its tensile strength (~ 2.6 MPa) and strain (~ 198%) in the radial direction, respectively. The high mechanical strength of all-wood hydrogels is mainly attributed to the strong hydrogen bonding, physical entanglement, and van der Waals forces between lignin molecules, cellulose nanofibers, and PVA chains. Thanks to its excellent flexibility, good conductivity, and sensitivity, the all-wood hydrogel can accurately distinguish diverse macroscale or subtle human movements, including finger flexion, pulse, and swallowing behavior. In particular, when “An Qi” was called four times within 15 s, two variations of the pronunciation could be identified. With recyclable, biodegradable, and adjustable mechanical properties, the all-wood hydrogel is a multifunctional soft material with promising applications, such as human motion monitoring, tissue engineering, and robotics materials.

**Supplementary Information:**

The online version contains supplementary material available at 10.1007/s40820-022-00827-3.

## Highlights

- An all-wood hydrogel was synthesized via a simply Hofmeister effect without the use of any chemical cross-linking agent.
- The all-wood hydrogel shows a high tensile strength of 36.5 MPa, a strain up to ~ 438%, and good conductivity, and can accurately distinguish diverse large or subtle human movements.
- The all-wood hydrogel has good recyclable, biodegradable, and adjustable mechanical properties.

**Supplementary Information:**

The online version contains supplementary material available at 10.1007/s40820-022-00827-3.

## Graphical abstract

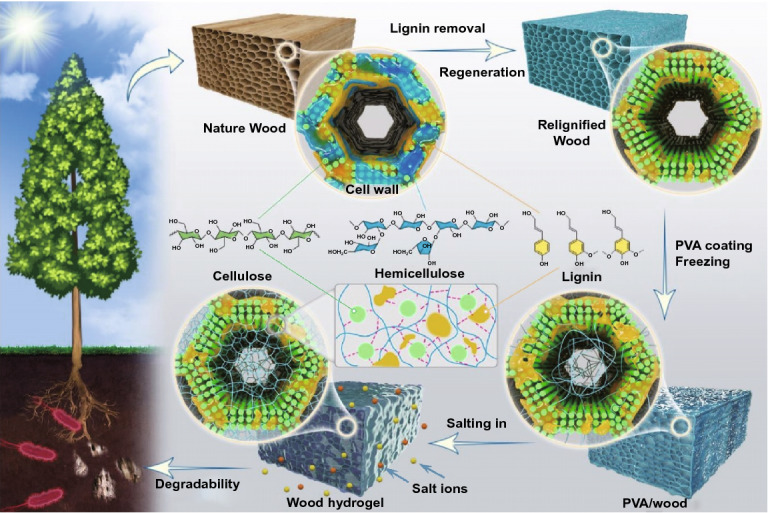

## Introduction

Tough and flexible hydrogels have attracted wide attention due to their potential applications in the fields of tissue engineering, implantable electronic devices, soft robots, sensors, drug delivery, etc*.* [1–5]. However, there are still some tricky problems needed to be solved in practical applications. For example, the loose cross-links and high water content of the synthetic hydrogels make them mechanically weak and unable to perform the actual tasks [6–8]. Although significant advancements have been made in enhancing the mechanical strength by adding composite fillers or forming 3D networks, these results are still not satisfactory compared with natural materials [9–11]. Besides, in most practical applications, the dynamic conversion of hydrogels between tough and soft states, or tough and dissolve (or degradation) states is highly necessary [12, 13]. For example, bioprobes should be designed to be rigid initially for easy insertion and flexible once they enter biological tissues [14–16]. Significant advances have been made in the use of structural engineering methods, such as the creation of homogeneous tough hydrogels by self-assembly and double network formation or anisotropic tough hydrogels by directional freeze casting and mechanical stretching [17–20].

Recently, a composite hydrogel containing a natural micro-/nanofiber reinforcer was explored, which significantly improved the mechanical properties of hydrogels [21–23]. Typically, natural wood can be used as support material after releasing the tight connections between cellulose fibers, generating tough wood structure hydrogels for enhanced mechanical strength, super-ion transport, or pressure sensors [10, 24, 25]. Natural wood is usually valued because of its highly anisotropic structure; however, the hard crystalline structure of cellulose makes it difficult to make the wood hydrogel flexible [26, 27]. Compared with homogeneous hydrogels, the strength of wood hydrogels is significantly increased, but even in their softest state, they are still not soft enough to match natural biological tissue [28–30]. Besides, during the wood processing, lignin with many reactive groups (e.g., methoxy and phenolic hydroxyl groups), as the second abundant biopolymer in natural plants, is generally discarded as waste, resulting in a serious waste of resources [31–34]. Furthermore, existing approaches have mainly focused on optimizing the micro-/nanostructures of hydrogels. However, the preparation of tough and flexible wood-based hydrogels with complex hierarchical structures using common natural polymers is still challenging.

**Fig. 1:**
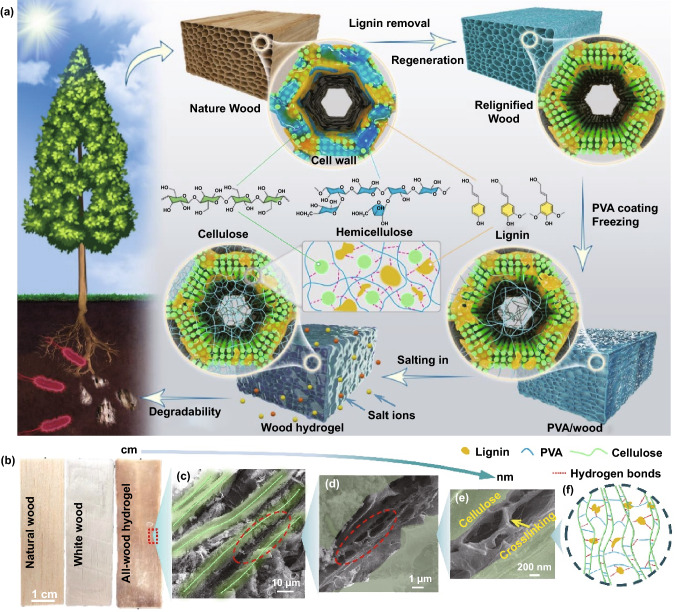
Fabrication process of the PVA-reinforced all-wood hydrogel. **a** The preparation process of the hydrogels via the Hofmeister effect includes the delignification, relignification, PVA soak process, freezing, and salt-assisted aggregation. **b** Digital images of natural wood, white wood, and all-wood hydrogel. **c–e** SEM images showing the hierarchical structure of the all-wood hydrogel. **f** Schematic diagram of the chemical cross-linking of cellulose fibers, lignin molecules, and PVA molecular chains

Different salts show distinguishable abilities to precipitate proteins/polymers from aqueous solutions, which is defined as the Hofmeister effect [35–37]. Some reports discussed the relationship between various ions and the solubility of the polymers (e.g., poly(vinyl alcohol) (PVA)) and made it possible to use different ions to obtain hydrogels with tunable mechanical properties [36, 38]. With these considerations and inspirations from the ion-specific phenomena, we design a PVA-reinforced all-wood hydrogel by constructing microstructure between cellulose fibers, PVA chains, and lignin molecules using the Hofmeister effect without modifications for the raw materials and any chemical cross-linking agent (Fig. 1a). After the processing, we have made the transition from macroscopic natural wood to nanomaterial (Fig. 1b–e). In this system, the cellulose fibers act as an aligned high-strength skeleton, PVA chains as the filler, and lignin molecules as the cross-linking agent (Fig. 1f). Therefore, a highly flexible and conductive all-wood hydrogel was prepared by a series of lignin rearrangements, PVA impregnation, and sodium sulfate salting, which is capable of accurately distinguishing various macroscopic or subtle human actions, including finger flexion, pulse, and swallowing behavior. With good flexibility, high mechanical strength and conductivity, good biodegradability, and recyclability, all-wood hydrogels have great potential for various advanced applications such as wearable sensors, soft robotics, tissue engineering, and drug delivery.

## Experimental Section

### Materials

Balsa wood was bought from JU FASHION Co. Ltd., China, and cut to the dimensions of 1 × 20 × 60 mm^3^. Sodium hydroxide (NaOH), sodium chlorite (NaClO_2_) (analytical grade, Sigma-Aldrich), poly(vinyl alcohol) (molecular weight Mw = 8.9 × 10^3^–9.8 × 10^3^ g mol^−1^, Aladdin), and sodium sulfate (analytical grade, Aladdin) were used as received. Deionized (DI) water was used for the preparation of all solutions.

### Delignification of the Balsa Wood

The balsa wood (1 × 20 × 60 mm^3^) was boiled in a 2.5 M NaOH solution using a 1-L reactor at 120 °C for 2 h. The separated solid wood from the brown mixture was transferred to 1 wt% NaClO_2_ solution (pH ~ 4.6) and boiled at 140 °C for 4 h to make it white enough (denoted as “white wood”). Then, the white wood was washed with ethanol and deionized water, respectively, and dried at -50 °C for 24 h. Besides, the brown mixture was filtered with HCl solution (pH < 2), centrifuged, and washed with deionized water to obtain alkali lignin.

### Relignification of the White Wood

The relignified wood was prepared by immersing the white wood in the 1,4-dioxane solution (20 mL) of alkali lignin (0.03 g). After immersing for 1 h, the wood sample was dried naturally at room temperature. The above procedure (immersing and drying) was repeated at different times to get relignified wood with different lignin contents. The detailed component of wood hydrogels is listed in Table S1.

### Preparation of the PVA and Salt Solution

The PVA solutions (5, 10, and 15 wt%) were prepared by dissolving PVA powder in deionized water at 90 °C. After 1 h of ultrasound treatment, a clear solution was obtained. The sodium sulfate solution (0.5, 1.0, 1.5, and 2.0 M) was prepared by dissolving in deionized water at room temperature. After 0.5 h of ultrasound, a clear solution was obtained.

### Preparation of the All-wood Hydrogel

For preparing an all-wood hydrogel, relignified wood (cellulose and alkali lignin) or white wood was immersed into the PVA solution and heated at 90 °C in an oil bath for 12 h. Subsequently, the container was frozen at − 20 °C. Finally, the sample was immersed into the sodium citrate solution for gelation at room temperature. For preparing pure PVA hydrogel, the PVA solution was frozen at − 20 °C and then immersed into the sodium citrate solution at room temperature. In contrast, other hydrogels with different lignin content, PVA content, sodium citrate concentration, and salting-out time were all prepared. The detailed formulation of each component is listed in Table S2.

### Characterization

The chemical structure of the lignin was analyzed by ^31^P nuclear magnetic resonance spectroscopy (NMR, AS 400, Zhongke-Niujin, China) and 2D NMR spectra (Bruker Avance III, Germany). The chemical structure of the all-wood hydrogel was also characterized using Fourier transform infrared spectroscopy (FT-IR). It was also performed by X-ray diffraction (XRD, Rigaku Ultima IV, Japan) with a 10° ~ 90° (2θ) scanning range and a speed of 2 s per degree. The microstructure images of the samples were carried out using a scanning electron microscope (FE-SEM, SUPRA 55VP, ZEISS, Germany) observing an SE2 pattern and 15 kV. 2D SAXS images of the all-wood hydrogel were obtained at the Bruker NANOSTAR of the Ceshigo Research Service, Shanghai, China. Confocal microscopy (Leica DMIL, Germany) was used to estimate the route of PVA into cellulose nanochannels of wood. Fluorescein sodium was used as a stain to premix in a PVA solution and then to prepare hydrogel using the above steps. The fluorescence was then observed. The tensile strength of samples was performed by an Instron 4465 instrument at room temperature. The hydrogel was coated with olive oil to reduce water loss and tested at a stretching speed of 1 cm min^−1^. The sensing test was performed by an LCR meter (TH2829A, Tonghui, China). The all-wood hydrogel was simply covered by two copper foils and glued onto the human skin using adhesive tape. Subsequently, the simple device was connected by the precision LCR meter (TH2829A, Tonghui, China) to record the signal changes.

## Results and Discussion

### Fabrication of the All-wood Hydrogel

**Fig. 2:**
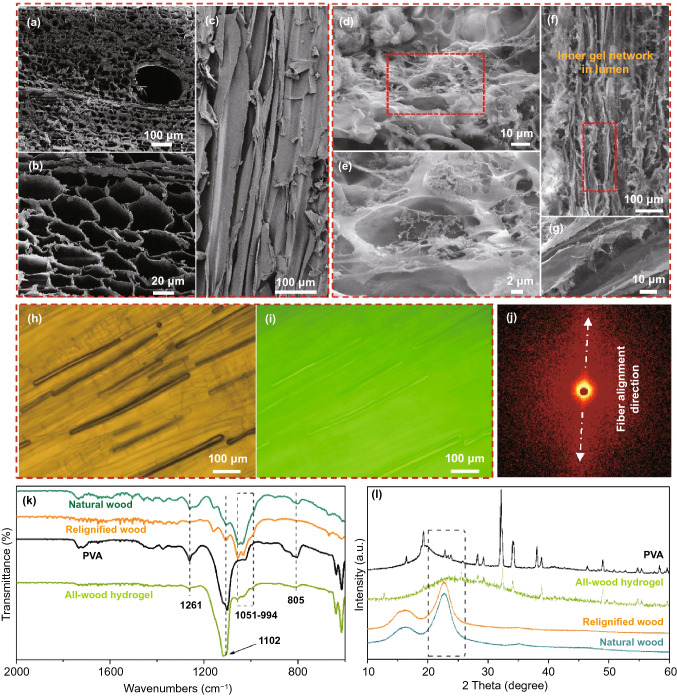
Morphological and structural characterizations of natural wood and all-wood hydrogels. SEM images of the natural wood: **a** the top-view SEM image and **b** its magnified image, and **c** the side-view SEM image of natural wood. SEM images of the all-wood hydrogel: **d** the top-view SEM image and **e** its magnified image, **f** the side-view SEM image and **g** its magnified image. **h**, **i** Confocal images showing the penetration of PVA into the microchannels of the all-wood hydrogel. Scale bar, 100 μm. **j** SAXS pattern of the prepared all-wood hydrogel. **k** FTIR spectra and **l** XRD patterns of the PVA, relignified wood, and all-wood hydrogel

We have successfully synthesized the all-wood hydrogel via a simply Hofmeister effect without the use of any chemical cross-linking agent. The starting wood material composed of cellulose, lignin, and hemicellulose has a cellular structure with numerous open nanochannels along the growth orientation (Fig. 2a–c). In the delignification process, most of the lignin can be extracted in situ using a mixture solution of NaOH and Na_2_SO_3_, and then the residual lignin can be further removed to obtain white wood by using a NaClO_2_ solution (pH ~ 4.6) (Fig. S1). Subsequently, the lignin regeneration process involves filling the micro-/nanochannels and interconnected nanopores of white wood with dissolved lignin (1,4-dioxane) (Table S1). In the delignification and relignification process, the original microscopic channels and chemical structure of natural wood were well maintained. After these treatments, many highly active hydroxyl groups in the cellulose fibers are exposed, but the mechanical strength of the wood becomes weaker.

To reconstruct the loose and porous structure of the relignified wood, filling the internal nanochannels with polymers is a preferred strategy. Thus, biodegradable PVA (a commonly used polymer) was introduced into the system [39–41]. PVA with rich hydroxyl groups can efficiently reconstruct wood by forming hydrogen bonds with cellulose fibers and lignin molecules. In our strategy, the relignified wood is immersed into the PVA solution at 90 °C for 24 h to reach full infiltration, which can be used to soften the cellulose fibers. We subsequently froze the sample at − 20 °C for 6 h, and then removed and immersed it in the 1.5 M Na_2_SO_4_ solution for gelation at room temperature. The obtained hydrogel shows an obvious interconnected network, which is attributed to the rich active groups of cellulose fibers, PVA chains, and lignin molecules (Fig. 2d–g). Note that the cellulose fibers in the hydrogel are originally part of the wood cell wall. When the above operation is repeated with the fluorescein disodium salt-labeled PVA, the confocal images clearly show that PVA solution can successfully enter into the internal nanochannels of cellulose fibers (Fig. 2h, i). When PVA-soaked and freeze-dried, the PVA chains become internal gel inside the cell lumen, which plays a critical role in maintaining the flexibility of the all-wood hydrogel. Besides, the all-wood hydrogel shows an obvious anisotropic structure (Fig. 2j), mainly because cellulose fibers retain the original structure of natural wood and act as the framework in the gel system [42–46].

To further investigate the chemical evolution during the all-wood hydrogel formation, the samples were characterized by FT-IR spectroscopy. Compared to the characteristic peaks of the natural wood, no significant new peaks appeared in the relignified wood (Fig. S2). The prepared all-wood hydrogel features absorption peaks at 1261 cm^−1^ for CH–OH stretching of tertiary alcohol in PVA, 1102 cm^−1^ for C–OH stretching vibration, 1051–994 cm^−1^ for C–H stretching, and 805 cm^−1^ for C–H bending, respectively (Fig. 2k). The FT-IR results indicated the formation of the cross-linking network in the system. Meanwhile, XRD patterns of the natural wood, delignified wood, and relignified wood exhibit similar diffraction peaks with cellulose I crystalline structure, suggesting its preservation after these treatments (Fig. S3). In addition, the XRD pattern of the all-wood hydrogel not only preserved the crystal structure of cellulose I, but also showed PVA characteristic peaks, confirming that the well-filled PVA gel entered the wood nanochannel (Fig. 2l). Besides, we also collected the chemical structure of lignin by 2D HSQC NMR spectra to explore the formation mechanism of the hydrogel.

**Fig. 3:**
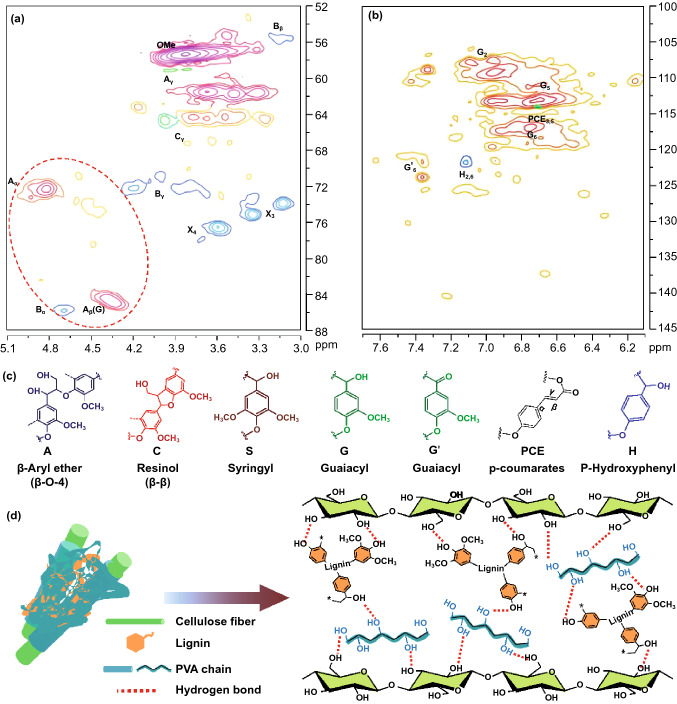
The chemical structure properties of the lignin. The NMR spectra of regenerated lignin: **a** aliphatic regions (*δ*_C_/*δ*_H_ 52–88/3.0–5.1), and **b** aromatic regions (*δ*_C_/*δ*_H_ 100–145/6.1–7.7). **c** The functional groups contained in the lignin molecules. **d** The structural linkage between cellulose fibers, lignin molecules, and PVA chains

We also tested the ^1^H-^13^C NMR spectra of the lignin in the aliphatic regions (*δ*_C_/*δ*_H_ 52–88/3.0–5.1) and aromatic regions (*δ*_C_/*δ*_H_ 100–145/6.1–7.7), as shown in Fig. 3a, b. The lignin contains some typical substructures including the *β*-O-4 alkyl-aryl ether, phenylcoumaran, syringyl, and guaiacyl-OH, and G-type units, etc*.* (Fig. 3c). The presence of a large number of -OH groups (e.g., phenolic-OH and Cx-OH) promotes the formation of entanglements between lignin, cellulose, and PVA chains through hydrogen bonding, resulting in a highly cross-linked network of all-wood hydrogels. Figure 3d schematically illustrates the interaction between cellulose nanofibers, lignin molecules, and PVA chains. Lignin molecules containing a large number of hydroxyl groups can form tight interactions with the hydroxyl groups in cellulose nanofibers and PVA molecular chains through hydrogen bonds, physical entanglement, and van der Waals forces, resulting in all-wood hydrogels with excellent mechanical properties. In contrast, the network of lignin-free hydrogels is formed only by the interaction of cellulose nanofibers with PVA molecular chains, and the physical entanglement between molecules is greatly reduced (Fig. S4). In short, the results show that the lignin, PVA, and cellulose all played critical roles in the high tensile strength of the all-wood hydrogel.

### Simultaneous Strengthening and Toughening of All-wood Hydrogels

**Fig. 4:**
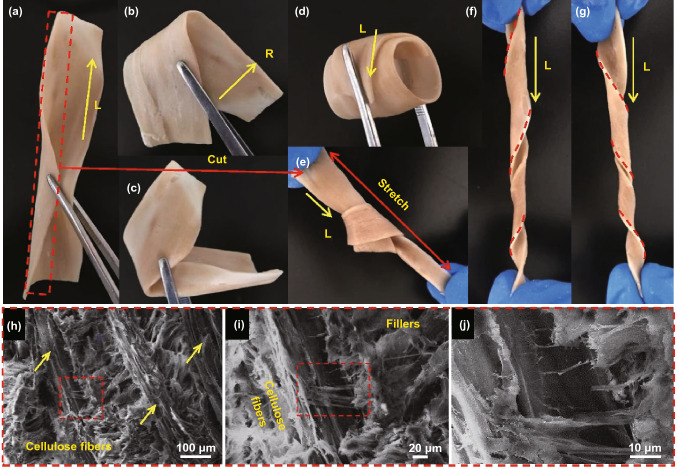
Digital images of the all-wood hydrogel (1 mm thick) folded in the **a** radial direction, **b** longitudinal direction, **c** in random directions, **d** wrapped, **e** stretched in the knotted case and curled to the **f** right and **g** left along to the longitudinal direction. **h-j** SEM images showing the cross-linking of all-wood hydrogels. Note that the cellulose nanofibers are tightly cross-linked to each other

After delignification, relignification, and gelation processes, the cellulose fibers in the gel network swell and soften, making the all-wood hydrogel highly flexible. Figure 4 shows the digital images and microscale morphology of the all-wood hydrogel under different stresses. Experimental results indicate that the all-wood hydrogels (1 mm thick) can be easily folded in the radial and longitudinal directions without rupture (Fig. 4a–c). Interestingly, it can also curl in the longitudinal direction, indicating that the cellulose fibers have softened sufficiently (Fig. 4d). Furthermore, we knotted a strip of all-wood hydrogel along the cellulose fibers direction. The stretched knot and curly ribbons further confirm the super-flexibility of the all-wood hydrogel (Fig. 4e–g). In particular, we can use this method to prepare flexible all-wood hydrogels, even with strips of wood up to 3 mm thick (Fig. S5). The partially open microchannels create space in the all-wood hydrogel to accommodate various deformations in an accordion-like manner, allowing the material to bend and withstand severe deformation without cracking. This unique crumpled, shrunken cell wall structure contributes to the excellent flexibility of the all-wood hydrogel, as shown in Fig. 4h–j and S6. The excellent mechanical properties of all-wood hydrogels are mainly attributed to the strong hydrogen bonding, physical entanglement, and van der Waals forces between cellulose nanofibers, lignin molecules, and PVA chains, and the toughening effect of cellulose nanofibers.

**Fig. 5:**
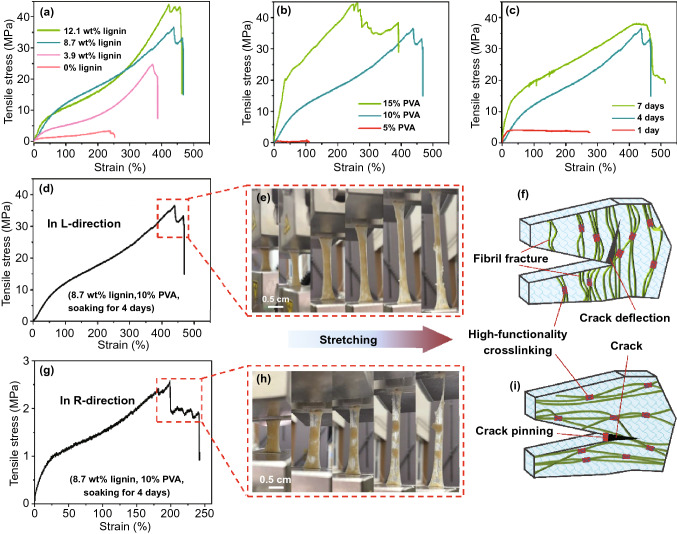
The mechanical strength and cross-linking mechanism of the all-wood hydrogel. The effect of **a** lignin content, **b** PVA content, and **c** salting time in the 1.5 M sodium sulfate solution on the tensile strength of the all-wood hydrogel in the L-direction, respectively. **d** The tensile stress–strain curves for all-wood hydrogel in the L-direction and **e** its fibrotic fracture during tension, and **f** the tensile mechanism at this state. **g** The tensile stress–strain curves for all-wood hydrogel in the R-direction and **h** its fibrotic fracture during tension, and **i** the tensile mechanism at this state. Here, the samples contain 10% PVA, soaking for 4 days, and different content of lignin in (a); the samples contain 8.7 wt% lignin, soaking for 4 days, and different content of PVA in (b); and the samples contain 8.7 wt% lignin, 10% PVA, and different salting time in (c). Besides, the samples are composed of 10 wt% PVA, 8.7 wt% lignin, and soaking for 4 days in (d, g)

Induced by the Hofmeister effect, crystal domains and numerous hydrogen bonds are formed in the hydrogel network, resulting in the high mechanical strength of the all-wood hydrogels [35, 38, 47–49]. The effects of lignin content, PVA content, and salting time on the tensile strength of all-wood hydrogels in the L-directional are further investigated. The tensile strength of the all-wood hydrogel enhances gradually with the increase in lignin concentration (Fig. 5a). In contrast, the white wood hydrogel without lignin has a weak tensile strength and exhibits significant breakage under external tension (Fig. S7). The results show that lignin can act as an efficient cross-linker in the hydrogel network, which significantly improves the mechanical properties of the hydrogel. Besides, the PVA concentration also has a great effect on the tensile strength of the all-wood hydrogel (Fig. 5b). The ultimate stresses were 0.5, 36.5, and 44.5 MPa, and the corresponding ultimate strains were 110%, 468%, and 393% after salting the all-wood hydrogels in 1.5 M sodium sulfate solution at room temperature for 1, 4, and 7 d, respectively. Due to the high viscosity and polymerization degree, the 15% PVA solutions cannot fully impregnate into the cellulose micro- and nanochannels. Furthermore, both tensile stress and tensile strain are enhanced with the increase in salting time and sodium sulfate concentration (Figs. 5c and S8). This is mainly attributed to the Hofmeister effect, i.e., the change in the aggregation state of PVA under the influence of sodium sulfate.

We also systematically investigate the anisotropic mechanical properties in the L- and R-directions of the all-wood hydrogel. The prepared all-wood hydrogel consists of 10 wt% PVA and 8.7 wt% lignin with a salting time of 4 days. The results show that the all-wood hydrogel has good longitudinal tensile properties with an ultimate stress of 36.5 MPa and an ultimate strain of 468%, both of which are much higher than that of previously reported cellulose-based hydrogels (Fig. 5d) [10, 30, 46, 50]. Notably, we can observe two significant breaks in the stress–strain curve of the all-wood hydrogel in the longitudinal direction (from 438 to 468%). The first break corresponds to a molecular chain break inside the nanocellulose with low ductility, while the second break is caused by a molecular chain break inside the PVA (Fig. 5d, red line). On a macro level, it is a gradual failure mode featuring stepwise fracture, which is a typical feature of anisotropic materials (Fig. 5e). During the stretching process along the L-direction, irregular faults are formed inside the hydrogel after the cellulose with fewer ductility breaks, which eventually overcomes the energy dissipation and leads to the hydrogel fracture with a crack deflection (Fig. 5f). In sharp contrast, the tensile strength of the lignin-free hydrogel is one order of magnitude lower (~ 4.7 MPa) than that of the all-wood hydrogel. When stretched along the L-direction, the lignin-free hydrogel exhibits an obvious stepwise fracture feature (Fig. S9).

Unlike the stretch strength along the L-direction, the all-wood hydrogel shows much weak toughness stretching with less than 2.6 MPa tensile strength and corresponding 198% strain in the R-direction (Fig. 5g). When stretched along the R-direction, the all-wood hydrogel mainly overcomes the cross-linking bonds between the cellulose, lignin, and PVA chains to cause the rupture, but scarcely involve the fracture of rigid cellulose chains. This result also corresponds to the super-long tail peak during the fracture process 198% to 242% of PVA (Fig. 5g, red line). As shown in Fig. 5h, the cellulose fibers can break easily because of the weak interaction forces of each cellulose fiber, leaving PVA gel to contribute a large tail peak. When stretched in the R-direction, the hydrogel overcomes the high-functionality cross-linking and occurs almost regular fracture instead of a crack deflection along a direction perpendicular to the cellulose fibers (Fig. 5i). Results indicate that the prepared all-wood hydrogel features obvious anisotropic properties, corresponding to the SAXS pattern in Fig. 2j.

### Sensing Performance of the All-Wood Hydrogel

**Fig. 6:**
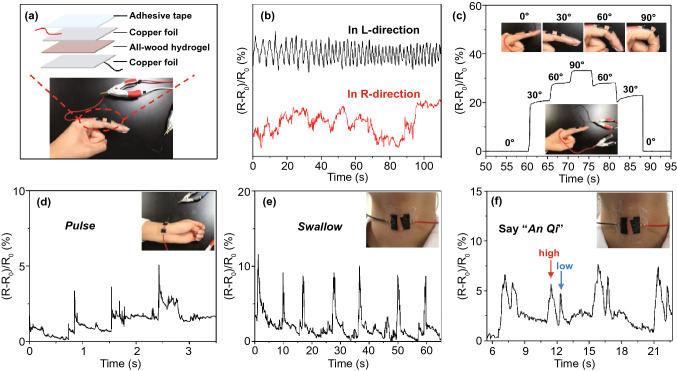
Signal change of all-wood hydrogel (with 8.7 wt% lignin, 10% PVA, 4 days for salting in sodium sulfate solution at room temperature) for real-time monitoring of various human motions. **a** Assembly process of the all-wood hydrogel sensor. **b** Real-time signal changes ((*R* − *R*_0_)/*R*_0_) of all-wood hydrogels bent repeatedly in the longitudinal and radial directions. The hydrogel can be used to monitor subtle muscle movements in real time, such as **c** finger flexion, **d** pulse beats, **e** swallowing, and **f** throat vibrations, as when a person says “An Qi” four times in a row

As previously reported on wood-hydrogel conductors, the cellulose nanofibers commonly act as nanochannels for ion transport [10, 25, 30, 50]. The strain sensitivity of the all-wood hydrogel is tested by calculating the gauge factor (GF, high value means higher sensitivity) of relative resistance variation ((R-R_0_)/R_0_) with strain. The curve can be divided into two linear responsive areas, including 0–108% region with a GF_1_ of 3.21 and 108–180% region with a GF_2_ of 6.16 (Fig. S10). Results indicate that the all-wood hydrogel has good strain responsiveness. To test the real-time monitoring capability of the all-wood hydrogel as a sensor, it was simply covered with two pieces of copper foil and taped to the human skin (Fig. 6a). Subsequently, the simple device is connected with the precision LCR meter to record the signal changes by repeatedly stimulating the assembled sensor device. The nanochannels in the well-aligned cellulose nanofibers act as a fast pathway for ion transport of the sensors. As shown in Fig. 6b, the fluctuation range of the current change values ((R-R_0_)/R_0_) in the longitudinal direction is smaller than that in the radial direction, indicating that the all-wood hydrogel has a significant anisotropic electrical sensitivity.

Due to the good conductivity, elasticity, and ductility of the all-wood hydrogel, it could be potentially applied as a sensor for real-time monitoring of various human motions. As illustrated in Fig. 6c, the all-wood hydrogel is sensitive enough to monitor the finger flexion at different angles ranging from 0 to 90°. The resistance signal exhibits great stability when maintaining the same finger bending, or bending and straightening the finger at the same angle, demonstrating the precise capture of instantaneous movements. Besides, the device can accurately distinguish subtle human movements, including pulse (Fig. 6d) and swallowing behavior (Fig. 6e). In particular, when “An Qi” was called four times within 15 s, two variations of the pronunciation could be identified (Fig. 6f). The results confirm that all-wood hydrogels have a strong ability to sense large/subtle body movements, making them very promising as wearable devices.

### Sustainability of the All-Wood Hydrogel

**Fig. 7:**
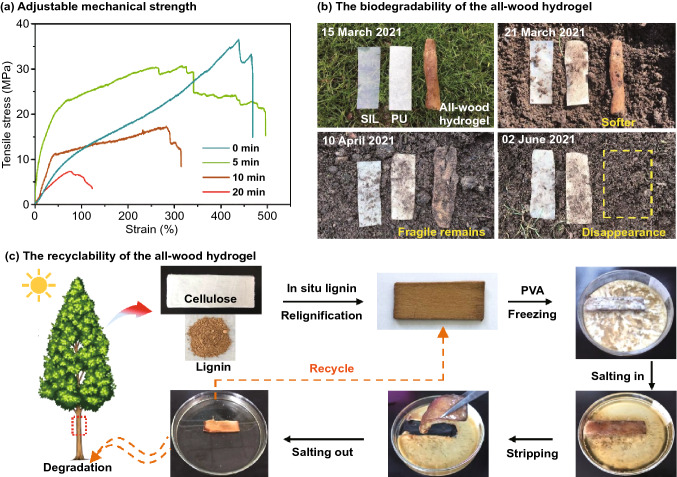
The mechanical tunability, biodegradability, and recyclability of all-wood hydrogels. **a** Changes in mechanical strength of the all-wood hydrogels after different salting times. **b** Biodegradability testing of the all-wood hydrogels and two commercially available silicone (SIL) and polyurethane (PU) adhesive films. **c** The recyclability of the all-wood hydrogel

According to the Hofmeister effect, the mechanical strength of the all-wood hydrogel can be effectively regulated as the hydrogel becomes soft or even dissolves when immersed in water. As shown in Fig. S11, the pure PVA gel prepared by the same method (also using 1.5 M Na_2_SO_4_ solution as the salt ion) was gradually dissolved within 30 min of immersion in deionized water [35]. The mechanical strength of the all-wood hydrogel decreased from 36.5 to 30.3, 17.2, and 7.3 MPa after salting out in deionized water for 5, 10, and 20 min, respectively (Fig. 7a). This is mainly due to the external diffusion of salt causing the PVA gel to dissolve in the framework. From a macroscopic point of view, it is a mode of progressive damage characterized by step fracture, while also maintaining its anisotropic character (Fig. S12). Note that all-wood hydrogels also decomposed after 2 h of immersion in deionized water (Fig. S13). Therefore, the mechanical strength of the all-wood hydrogel can be adjusted by controlling the immersion time of the all-wood hydrogel in deionized water.

We buried the all-wood hydrogel, silicone (SIL, a medical gel film), and polyurethane (PU film, a medical gel film) together in a 5–10-cm-deep soil (Fig. 7b). After a week of natural processes, the all-wood hydrogels became soft in the soil, mainly due to the diffusion of salt ions to the soil [33, 37]. The hydrogel then degraded into a brittle residue, which was mainly due to microbial corrosion. Finally, the cellulose and lignin of the all-wood hydrogel gradually degraded until they were all gone after another 2 months [51]. In contrast, SIL and PU films retained almost their original shape after the same burial time, indicating the non-biodegradability of these commonly used films. Besides, the all-wood hydrogel also exhibits good recyclability (Fig. 7c). The residual sample retained cellulose and lignin well after salting and can be soaked again with PVA to obtain a new all-wood hydrogel or discarded directly into nature to be degraded by microorganisms. We can also recover PVA from the solution after soaking.

## Conclusions

In conclusion, an all-wood hydrogel with good flexibility, conductivity, and tunable mechanical strength was successfully synthesized using a simple Hofmeister effect without modifying the raw material or using any chemical cross-linking agent. With aligned cellulose as the rigid backbone and PVA and lignin as the cross-linked network, the prepared all-wood hydrogel shows high tensile strength and good flexibility. Tensile tests results indicate the tensile strength of the all-wood hydrogel being 36.5 MPa and the L-direction strain being 438%, which was 14 times and ~ 2 times higher than the R-direction, respectively. In addition to the anisotropic mechanical strength, the conductivity of the all-wood hydrogels exhibited significant anisotropy due to the arrangement of the cellulose fibers. With high flexibility, high mechanical strength, and good conductivity, all-wood hydrogels can be used as sensors to detect a variety of large or small human movements, including finger flexion, pulse, and swallowing behavior. In addition, the all-wood hydrogel not only has tunable mechanical strength but also degrades very easily in the natural environment. This study demonstrates an effective, green, and recyclable method to prepare flexible and biodegradable wood-based hydrogels with tunable mechanical strength, paving the way for the construction of high-performance sustainable soft electronic materials.

## Supplementary Information

Below is the link to the electronic supplementary material.

Supplementary file1 (PDF 1116 kb)
