# Supplementary material for: Highly Flexible and Broad-Range Mechanically Tunable All-Wood Hydrogels with Nanoscale Channels via the Hofmeister Effect for Human Motion Monitoring
Source: Nanomicro Lett. 2022 Mar 29;14:84. doi: 10.1007/s40820-022-00827-3 (PMC8964865; doi:10.1007/s40820-022-00827-3)
Supplement: Supplementary file 1 — Supplementary file1 (PDF 1116 kb) [file 40820_2022_827_MOESM1_ESM.pdf]

Supporting Information for

Highly Flexible and Broad-Range Mechanically Tunable All-Wood Hydrogels with Nanoscale Channels via the Hofmeister Effect for Human Motion Monitoring

Guihua Yan[1], Shuaiming He[2,] *, Gaofeng Chen[1], Sen Ma[1], Anqi Zeng[1], Binglin Chen[1], Shuliang Yang[1], Xing Tang[1], Yong Sun[1], Feng Xu[3], Lu Lin[1,] *, and Xianhai Zeng[1,] *

[1] College of Energy, Xiamen University, Xiamen 361102, P. R. China

[2] State Key Laboratory of Pulp and Paper-making Engineering, South China University of Technology, Guangzhou 510640, P. R. China

[3] Beijing Key Laboratory of Lignocellulosic Chemistry, Beijing Forestry University, No. 35, Tsinghua East Road, Haidian, Beijing 100083, P. R. China

*Corresponding authors. E-mail: he.shuaiming@gmail.com (Shuaiming He), lulin@xmu.edu.cn (Lu Lin), xianhai.zeng@xmu.edu.cn (Xianhai Zeng)

Supplementary Figures and Tables

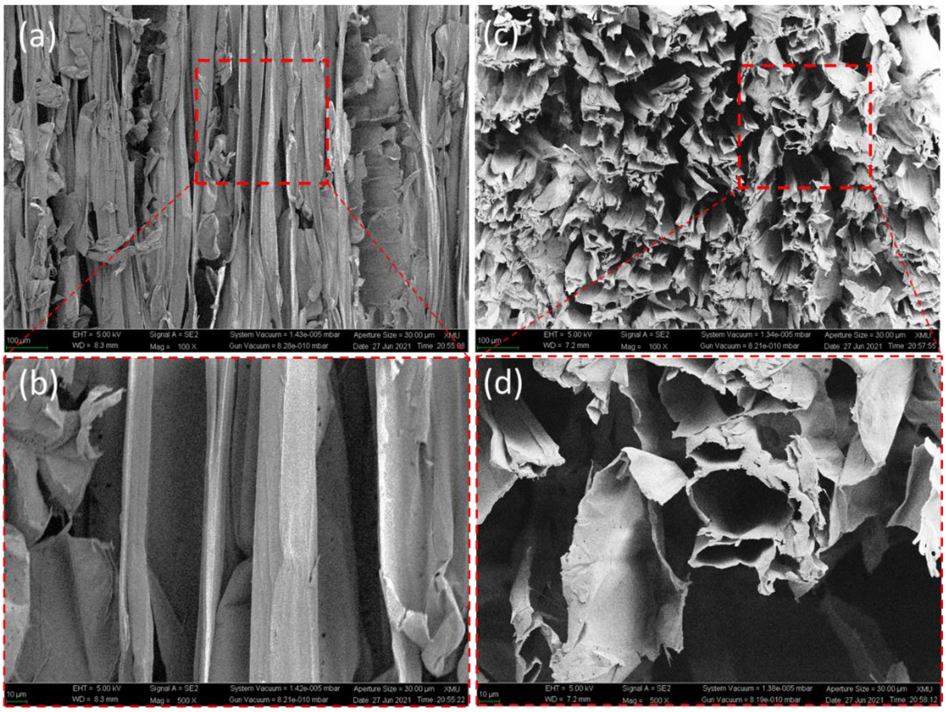

Fig. S1 Morphologies of the white wood from the **(a)** side-view and **(b)** its magnified image; and from the **(c)** top-view and **(d)** its magnified image. The SEM images show the loose structure between the cellulose fibers

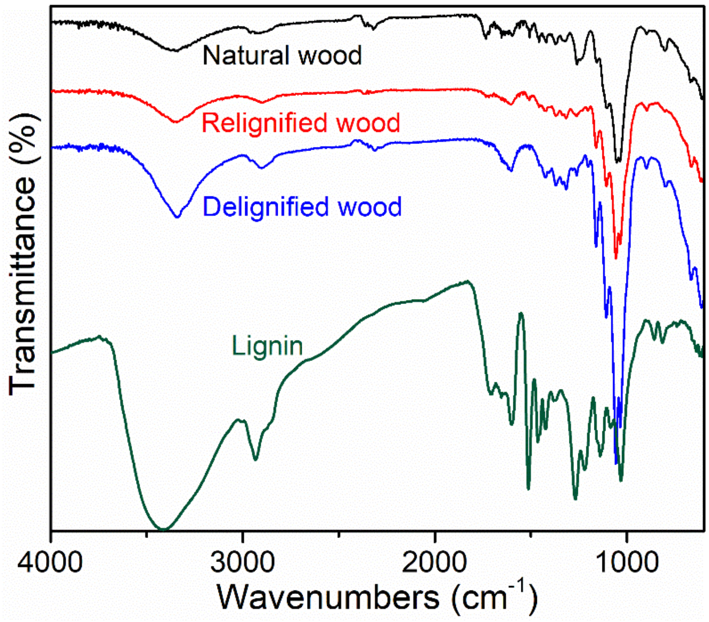

Fig. S2 FTIR spectra of the natural wood, delignified wood, and relignified wood

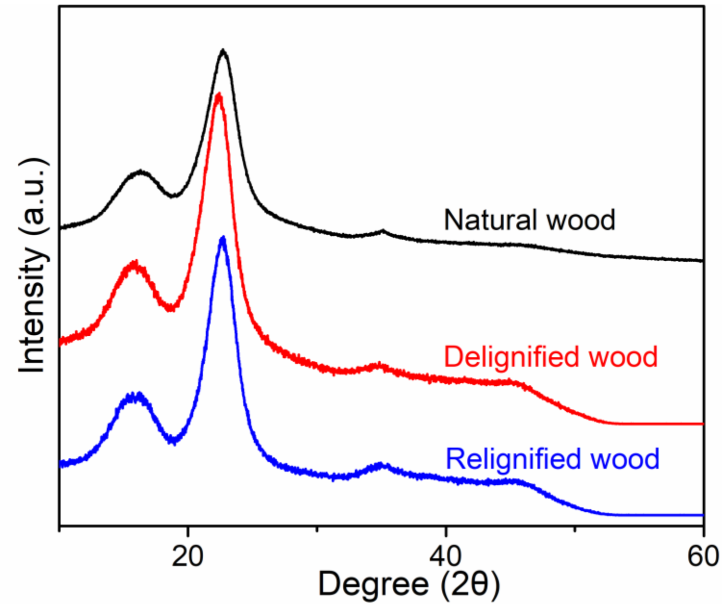

Fig. S3 XRD spectra of the natural wood, delignified wood, and relignified wood

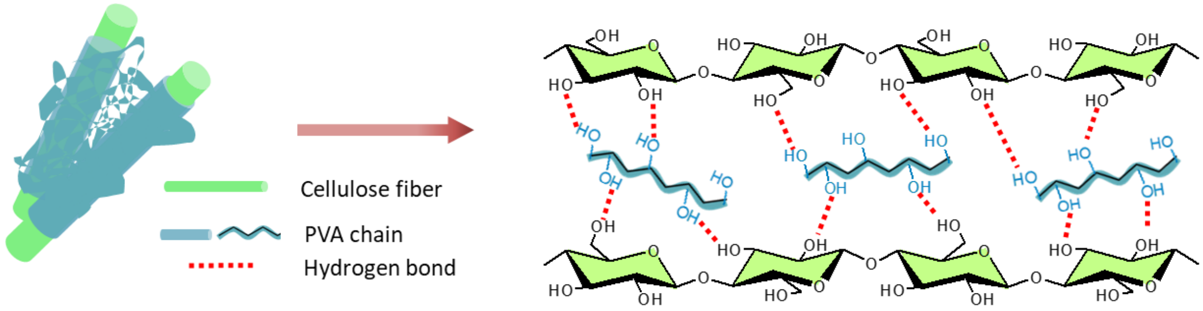

Fig. S4 The structural linkage of the white wood hydrogel between cellulose fibers and PVA chains

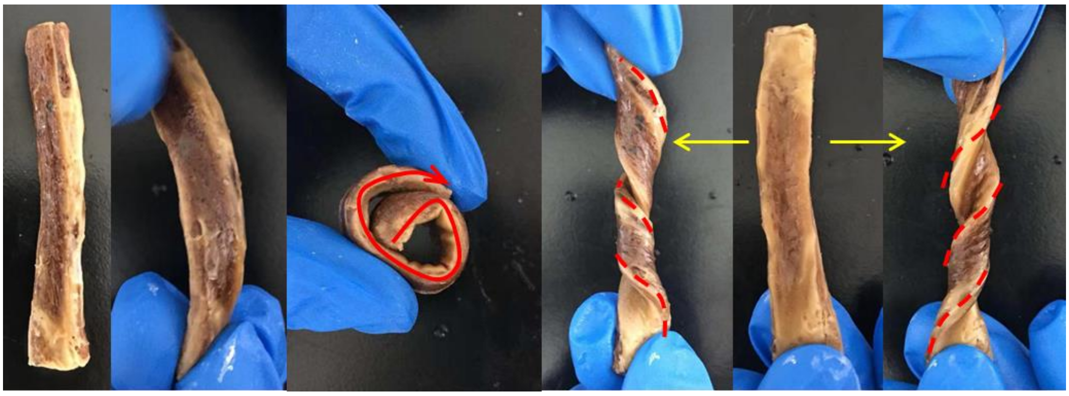

Fig. S5 Digital images of the all-wood hydrogel with a thickness of 3 mm

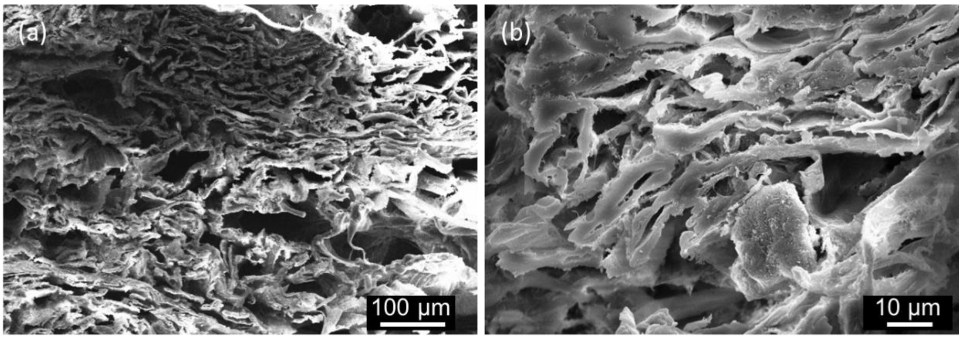

Fig. S6 Cross-section view of the all-wood hydrogel subjected to bending. Note that the partially open, crumpled cell wall structure in the all-wood hydrogel creates space to accommodate various deformations

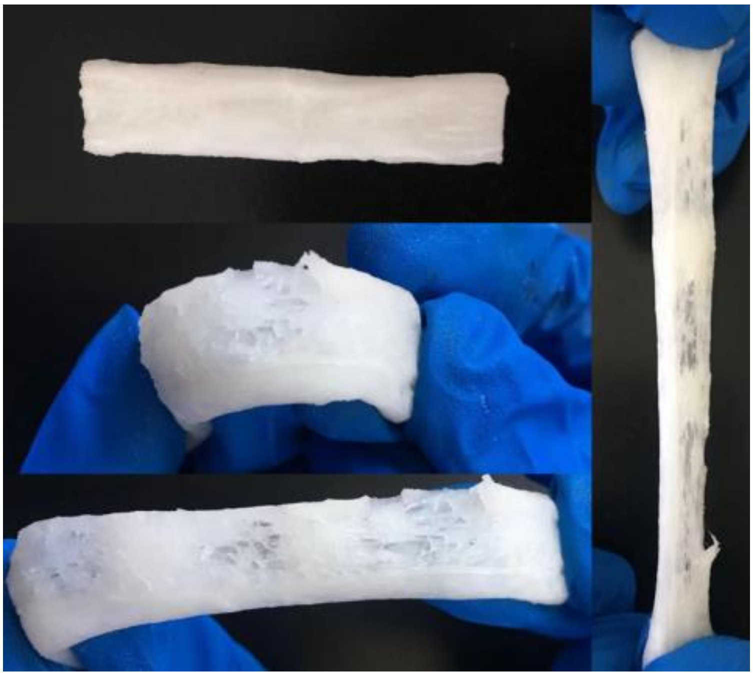

Fig. S7 Digital images of the white wood hydrogel. Note that the white wood hydrogel exhibited significant breakage under external tension

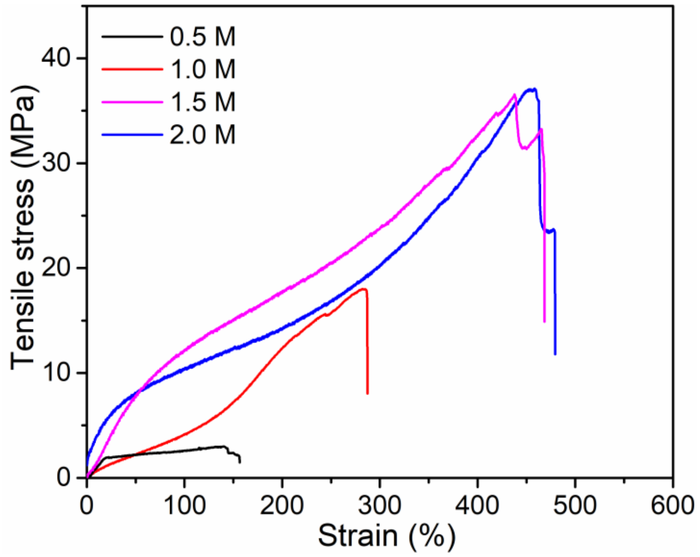

Fig. S8 The effect of sodium sulfate concentration on the tensile strength of the all-wood hydrogel

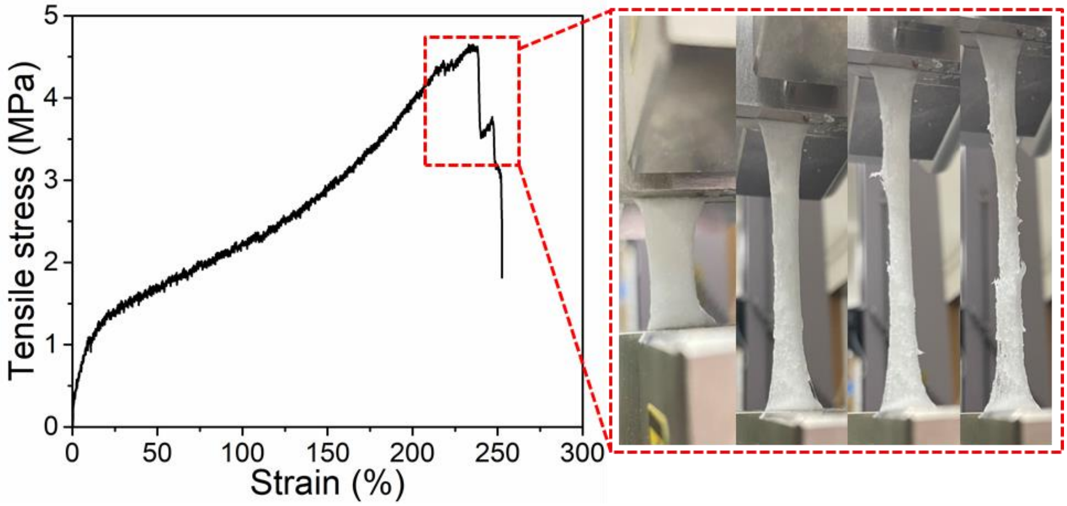

Fig. S9 The tensile strength of the white wood hydrogel without lignin

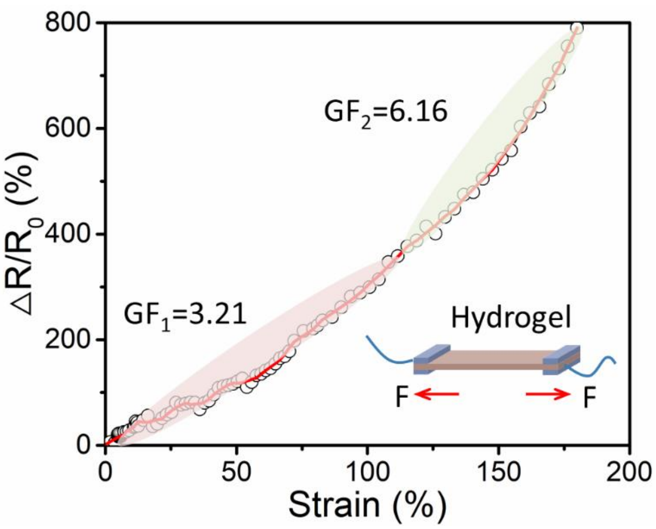

Fig. S10 Relative resistance variation curves of the all-wood hydrogel as the strain

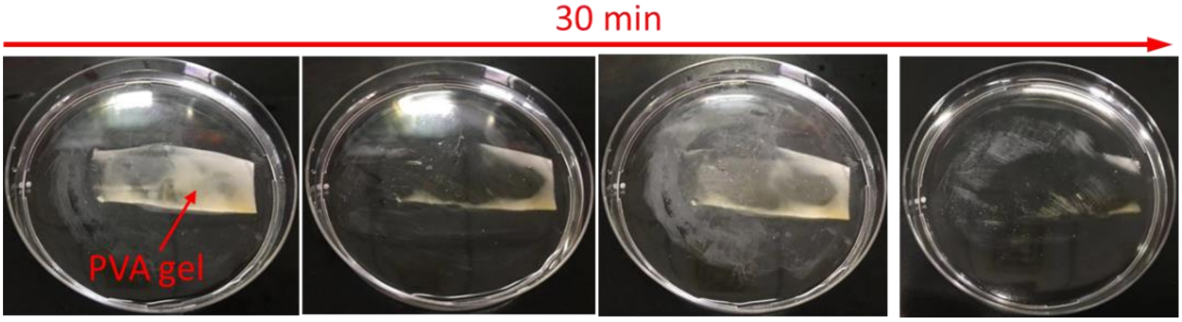

Fig. S11 The images of the PVA gel when salting out in the water for 30 min

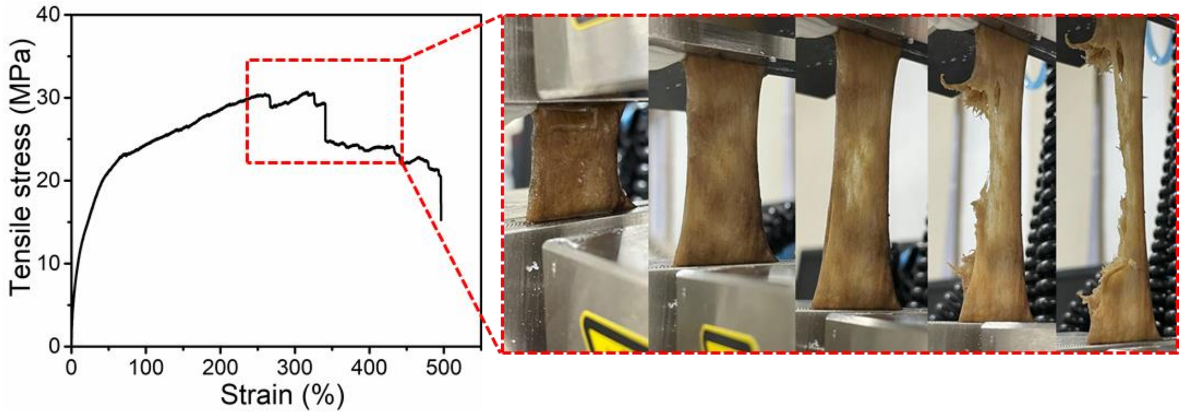

Fig. S12 The tensile strength of the all-wood hydrogel after 5 min of salting in water

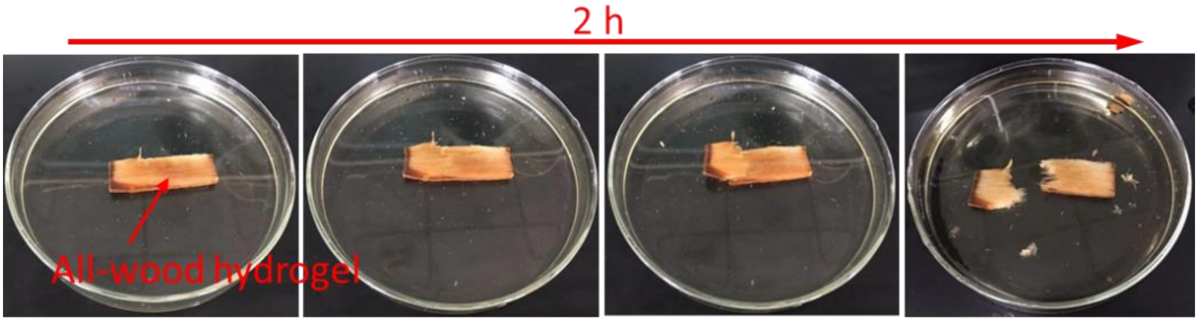

Fig. S13 The images of the all-wood hydrogel when salting out in the water for 2 h

Table S1 The chemical compositions of the all-wood hydrogel, including cellulose, hemicellulose, and lignin after resetting the lignin into the white wood for different times

Reset times of lignin	Cellulose (wt%)	Hemicellulose (wt%)	Lignin (wt%)
1	60.55	27.05	3.9
2	59.51	27.05	8.7
3	65.25	25.52	12.1

Notes: The lignin was obtained from the natural wood by delignification process. The collected lignin was then refilled into the microscopic channels of the white wood.

Table S2 Composition of various full-wood hydrogels with different lignin content, PVAcontent, Na_2SO_4 concentration, and salting time in the 1.5 M sodium sulfate solution

**Hydrogels**	**White wood (g)**	**Lignin/all-wood (wt%)**	**PVA (g)**	**Na_2SO_4 (mol L^{-1})**	**Salting time in Na_2SO_4 (days)**	**Water (mL)**
All-wood hydrogels	0.2	**3.9**	1.0	1.5	4	10
	0.2	**8.7**	1.0	1.5	4	10
	0.2	**12.1**	1.0	1.5	4	10
	0.2	8.7	**0.2**	1.5	4	10
	0.2	8.7	**0.5**	1.5	4	10
	0.2	8.7	**1.0**	1.5	4	10
	0.2	8.7	**1.5**	1.5	4	10
	0.2	8.7	1.0	**0.5**	4	10
	0.2	8.7	1.0	**1.0**	4	10
	0.2	8.7	1.0	**1.5**	4	10
	0.2	8.7	1.0	**2.0**	4	10
	0.2	8.7	1.0	1.5	**1**	10
	0.2	8.7	1.0	1.5	**4**	10
	0.2	8.7	1.0	1.5	**7**	10
White wood hydrogels	0.2	0	1.0	1.5	4	10
PVA hydrogels	0	0	1.0	1.5	4	10
